# The Therapeutic Monoclonal Antibody Bamlanivimab Does Not Enhance SARS-CoV-2 Infection by FcR-Mediated Mechanisms

**DOI:** 10.3390/pathogens12121408

**Published:** 2023-11-30

**Authors:** Robert W. Cross, Christopher M. Wiethoff, Patricia Brown-Augsburger, Shawn Berens, Jamie Blackbourne, Ling Liu, Xiaohua Wu, Jonathan Tetreault, Carter Dodd, Ramtin Sina, Derrick R. Witcher, Deanna Newcomb, Denzil Frost, Angela Wilcox, Viktoriya Borisevich, Krystle N. Agans, Courtney Woolsey, Abhishek N. Prasad, Daniel J. Deer, Joan B. Geisbert, Natalie S. Dobias, Karla A. Fenton, Beth Strifler, Philip Ebert, Richard Higgs, Anne Beall, Sumit Chanda, Laura Riva, Xin Yin, Thomas W. Geisbert

**Affiliations:** 1 Department of Microbiology and Immunology, University of Texas Medical Branch, Galveston, TX 77555, USAabnprasa@utmb.edu (A.N.P.);; 2 Galveston National Laboratory, University of Texas Medical Branch, Galveston, TX 77555, USA; 3 Eli Lilly and Company, Indianapolis, IN 46285, USA; brown-augsburger_patricia_l@lilly.com (P.B.-A.); berens_shawn_j@lilly.com (S.B.);; 4 Charles River Laboratories, Inc., Reno, NV 89511, USA; deanna.newcomb@charter.net (D.N.); angela.wilcox@crl.com (A.W.); 5 Immunity and Pathogenesis Program, Infectious and Inflammatory Disease Center, Sanford Burnham Prebys Medical Discovery Institute, 10901 North Torrey Pines Road, La Jolla, CA 92037, USA; 6 Department of Immunology and Microbiology, Scripps Research, La Jolla, CA 92037, USA

**Keywords:** bamlanivimab, antibody-dependent enhancement, SARS-CoV-2, COVID-19, monoclonal antibodies

## Abstract

As part of the non-clinical safety package characterizing bamlanivimab (SARS-CoV-2 neutralizing monoclonal antibody), the risk profile for antibody-dependent enhancement of infection (ADE) was evaluated in vitro and in an African green monkey (AGM) model of COVID-19. In vitro ADE assays in primary human macrophage, Raji, or THP-1 cells were used to evaluate enhancement of viral infection. Bamlanivimab binding to C1q, FcR, and cell-based effector activity was also assessed. In AGMs, the impact of bamlanivimab pretreatment on viral loads and clinical and histological pathology was assessed to evaluate enhanced SARS-CoV-2 replication or pathology. Bamlanivimab did not increase viral replication in vitro, despite a demonstrated effector function. In vivo, no significant differences were found among the AGM groups for weight, temperature, or food intake. Treatment with bamlanivimab reduced viral loads in nasal and oral swabs and BAL fluid relative to control groups. Viral antigen was not detected in lung tissue from animals treated with the highest dose of bamlanivimab. Bamlanivimab did not induce ADE of SARS-CoV-2 infection in vitro or in an AGM model of infection at any dose evaluated. The findings suggest that high-affinity monoclonal antibodies pose a low risk of mediating ADE in patients and support their safety profile as a treatment of COVID-19 disease.

## 1. Introduction

The COVID-19 pandemic caused by the novel severe acute respiratory syndrome coronavirus-2 (SARS-CoV-2) continues to have a profound effect on public health and economies globally. The scale of this pandemic has created an unprecedented demand for medical countermeasures including vaccines and therapeutics, such as monoclonal antibodies (mAbs). Therapeutic COVID-19 mAbs interact with the receptor-binding domain (RBD) of SARS-CoV-2 and inhibit spike protein attachment to human angiotensin-converting enzyme 2 (ACE2) receptors. This prevents viral entry into cells and viral replication, and results in potent virus neutralization [1,2,3].

Neutralization, however, is only one of several mechanisms by which antibodies can interfere with viral infection. While antibody effector functions play an important role in immunity against several viruses including HIV [4] and Ebola [5], some studies have also shown that at sub-neutralizing titers, anti-viral antibodies can lead to antibody-dependent enhancement (ADE) of viral replication and increased disease burden [6]. The most common mechanisms of ADE involve binding of opsonized viral particles to Fc gamma receptors (FcγR) on myeloid cells or complement receptors on a variety of cells, resulting in viral entry and productive replication in these lineages. The final outcome of ADE can be disease enhancement due to increased viral loads [7,8].

To date, ADE of viral replication and disease has been described for a variety of different viruses [9,10,11]. In the 1960s, both the respiratory syncytial virus vaccine and the measles vaccine failed to elicit long-lasting protective antibodies in pediatric cohorts. Upon virus exposure, the non-neutralizing antibody response in some vaccinated patients resulted in exacerbation of disease, largely due to immune complex deposition [9]. More recent epidemiological studies have shown that the presence of neutralizing antibodies to one strain of the dengue virus (DENV), elicited by prior infection or vaccination, resulted in enhanced disease upon a second infection with a different serotype [11]. In vitro studies have found that sub-neutralizing IgG antibodies facilitate the increased uptake of the IgG-DENV virion complex into FcγR-expressing cells resulting in productive replication and subsequent disease pathology [12,13]. Furthermore, convalescent plasma from patients recovering from DENV has been shown to enhance Zika virus infection in vitro, indicating the sero-cross-reactivity between the two flaviviruses [14]. The role of ADE in SARS-CoV-2 infection and its potential to exacerbate disease remains unclear [15]. Some in vitro studies have demonstrated that human coronavirus antibodies may enhance infection of SARS-CoV in ACE2-negative, FcγR-expressing cells [16,17,18,19].

Two possible mechanisms for FcγR-mediated ADE in SARS-CoV-2 infection have been proposed: firstly, mediated by antibody-dependent infection of macrophages via Fc receptors; and secondly, related to the activation and degranulation of mast cells with Fc receptor-bound SARS-CoV-2 antibodies, leading to increased histamine release [20]. One study showed FcγRIIA- and FcγRIIIA-mediated ADE of SARS-CoV-2 infection, but this did not affect proinflammatory cytokine production in monocyte-derived macrophages [21]. Another report showed increased FcγR-mediated infection by a pseudovirus construct in the presence of selected RBD and N-terminal domain (NTD) antibodies [22]. However, when tested in vivo in a cynomolgus model of SARS-CoV-2 infection, no clear evidence of ADE was observed in non-human primates (NHPs) [22]. Additionally, convalescent plasma from patients with severe COVID-19 disease and an S1 RBD mAb have been shown to enhance infection of SARS-CoV-2 in vitro by promoting virus–cell membrane fusion [23]. However, controlled studies in NHPs using both high and low doses of convalescent plasma showed no enhancement of disease [24]. Further, clinical studies also demonstrated no enhancement of COVID-19 disease for patients treated with convalescent plasma [25,26]. One recent study observed that patients with COVID-19 disease produced neutralizing antibodies against the NTD, as well as antibodies that enhanced SARS-CoV-2 binding to ACE2 [27]. Two recent reports did not find evidence of ADE by SARS-CoV-2 mAbs, neither in vitro nor in vivo [28,29].

The ability of therapeutic neutralizing mAbs to exacerbate COVID-19 through ADE in vivo remains unknown, and the risk of SARS-CoV-2 infection enhancement by vaccines or neutralizing mAbs warrants further research to ensure the safety of current and future therapies. Here, we studied the question of whether bamlanivimab, a COVID-19 mAb, holds the potential for ADE of SARS-CoV-2 infection in vitro and in an NHP model of COVID-19. Bamlanivimab was the first COVID-19 mAb to be granted Emergency Use Authorization (EUA) in November 2020 by the U.S. Food and Drug Administration (FDA). Lilly voluntarily asked the FDA to revoke the EUA for bamlanivimab in April 2021 due to the increase in SARS-CoV-2 viral variants resistant to bamlanivimab and not due to any new safety concerns [30]. To evaluate whether bamlanivimab mediates viral uptake and replication in immune cells, we evaluated viral replication in cell culture models using authentic virus. For our in vitro studies, we relied on FcR-expressing cells, primary human macrophages, THP-1 cells and Raji cells that were exposed to replicating SARS-CoV-2 and treated with a range of bamlanivimab concentrations. Furthermore, we investigated whether prophylactic treatment with bamlanivimab mediated ADE of SARS-CoV-2 in an animal model. Due to their physiological similarity to humans, NHPs are considered the gold standard for studying pathophysiological responses to infection and treatment efficacy, and African green monkeys (AGMs) support a high level of SARS-CoV-2 replication and develop respiratory disease similar to humans. Moreover, we and others recently described an AGM model of COVID-19 that recapitulates many salient features of mild-to-severe human disease [31,32,33,34]. For in vivo studies, AGMs were administered at either sub-saturating or saturating doses of bamlanivimab and challenged with an inoculum of SARS-CoV-2 virus. Viral replication coupled with gross and histological pathology in control and treatment groups was assessed to determine whether ADE had occurred.

## 2. Materials and Methods

### 2.1. In Vitro Methods

#### 2.1.1. Cell Culture

Vero-E6 cells (African Green Monkey kidney epithelial cell line permissive to SARS-CoV-2 infection) were cultured in minimum Essential Medium Eagle (MEM) with Earle’s salts and L-glutamine, 10% FBS, 1X MEM non-essential amino acids and 1X sodium pyruvate, and 1X penicillin-streptomycin. Raji (human B lymphoblastoid cell line), ST486 (human B lymphoblastoid cell line negative for FcγR) and THP-1 (human monocytic cell line) cells were propagated in RPMI 1640 medium with 2 mM L-glutamine and 10 mM HEPES, 10% heat inactivated FBS, and 1X penicillin-streptomycin. Primary human macrophages from three de-identified donors were procured from STEMCELL Technologies, Inc, thawed according to the manufacturer’s instruction and cultured in complete cell culture medium containing RPMI 1640 and 10% FBS.

The day before infection (Day 0), cells were seeded into 96-well plates (flat-bottom for adherent cells and V-bottom for suspension cells) at a density of ~4−5 × 10^4^ cells per well. SARS-CoV-2 (clinical isolate USA-WA1/2020) was procured from BEI Resources and propagated in Vero-E6 cells for two passages. The day after cell seeding (Day 1) SARS-CoV-2, in an amount corresponding to an MOI of 0.1 per well, was mixed with either bamlanivimab or an isotype control antibody at a concentration of 0, 0.3, 3, 30, 300 or 3000 ng/mL and incubated at 37 °C for 1 h.

Following the incubation of virus and antibody, cells (N = 3) were treated with the antibody–virus mixture and incubated at 37 °C for an additional 1 h to allow for viral infection of the cells. Cells were washed once with Dulbecco’s phosphate-buffered saline (PBS). Complete growth medium was replaced, and the cells were incubated at 37 °C for either 6 h or 48 h. At the indicated time point, cell culture supernatant was harvested. Both cells and supernatant were frozen at −80 °C for RNA extraction and qPCR analysis of viral RNA.

#### 2.1.2. Flow Cytometry

Cell surface FcγR expression on THP1, Raji, and ST486 cell lines, as well as human primary macrophages, were characterized by flow cytometry using fluorescence conjugated antibodies specific to FcγRI, FcγRIIa, FcγRIIb, or FcγRIII. Fluorescence-conjugated matched isotype controls were included in the studies as negative controls. Cells were transferred to a 96-well round bottom polypropylene plate and washed twice in cold AutoMACS running buffer then blocked with human TruStain FcX block in AutoMACS running buffer for 20 min on ice. Cells were subsequently incubated with FcγR-specific antibodies diluted in AutoMACS running buffer for 30 min on ice protected from light. Following this, cells were washed twice with AutoMACS running buffer and acquired using a BD LSRFortessa II flow cytometer (Becton Dickinson, Franklin Lakes, NJ, USA).

#### 2.1.3. RT-PCR

Intracellular and extracellular viral RNA was purified from infected cells and supernatants with the TurboCapture 384 mRNA Kit (Qiagen, Germantown, MD, USA) in accordance with the manufacturer’s instructions, respectively. The purified RNA was subjected to first-strand cDNA synthesis using the high-capacity cDNA reverse transcription kit (Applied Biosystems, Inc., Waltham, MA, USA) containing 2019-nCoV_N2 Reverse Primer (10 µM) for the positive strand, 2019-nCoV_N2 Forward Primer (10 µM) for the negative strand, or random primers for both strands. Real-time PCR (RT-PCR) analysis was performed using TaqPath 1-step RT-qPCR Master Mix (Applied Biosystems, Inc.) and ACTINB CTRL Mix (VCP reporter) (Applied Biosystems, Inc.) for housekeeping genes. The following primers and probe for qPCR measurements of viral genes were used: 2019-nCoV_N2 Forward Primer (5′-TTA CAA ACA TTG GCC GCA AA-3′); 2019-nCoV_N2 Reverse Primer (5′-GCG CGA CAT TCC GAA GAA-3′); and 2019-nCoV_N2 Probe (5′-FAM-ACA ATT TGC CCC CAG CGC TTC AG-BHQ1-3′). Samples were run on a ViiA 7 Real-Time PCR System (Applied Biosystems, Inc.). Absolute quantification of viral RNA (genome equivalent levels) was performed using a standard control that was simultaneously analyzed on the same plate. For all samples above the LLOQ of the assay, comparisons across multiple groups were performed by two-way ANOVA and Holm–Sidak pair-wise post-hoc analysis with a *p*-value of <0.05 considered significant. LLOQ was 10,000 copies/mL or 10,000 copies/g tissue. In figures, all data points found to below quantitative limits were assigned a value of LLOQ/2.

#### 2.1.4. Surface Plasmon Resonance

Biacore T100 (Cytiva, Marlborough, MA, USA), Biacore reagents, and Scrubber 2 Biacore Evaluation Software (Biologics 2008) were used for the SPR analysis of bamlanivimab binding. A series S CM5 chip (Cytiva P/N BR100530) was prepared using the manufacturer’s EDC/NHS amine coupling method (Cytiva P/N BR100050). Briefly, the surfaces of all four flow cells (FC) were activated by injecting a 1:1 mixture of EDC/NHS for 7 min at 10 μL/min. Protein A (Calbiochem P/N 539202) was diluted to 100 μg/mL in 10 mM acetate and pH 4.5 buffer, and immobilized for approximately 4000 RU onto all 4 FCs by 7 min injection at a flow rate of 10 μL/min. Unreacted sites were blocked with a 7 min injection of ethanolamine at 10 μL/min. Injections of 2 × 10 μL of glycine, pH 1.5, were used to remove any noncovalently associated protein. Running buffer was 1× HBS-EP+ (TEKNOVA, P/N H8022). The FcγR ECDs-FcγRI (CD64), FcγRIIA_131R, and FcγRIIA_131H (CD32a), FcγRIIIA_158V, FcγRIIIA_158F (CD16a), and FcγRIIb (CD32b) were produced from stable CHO cell expression. All FcγR ECDs were purified using IgG Sepharose and size exclusion chromatography.

For FcγRI binding, antibodies were diluted to 2.5 μg/mL in running buffer, and approximately 150 RU of each variant was captured in FCs 2 through 4 (RUcaptured). FC1 was the reference FC; therefore, no antibody was captured in FC1. FcγRI ECD was diluted to 200 nM in running buffer and then two-fold serially diluted in running buffer to 0.78 nM. Duplicate injections of each concentration were injected over all FCs at 40 μL/min for 120 s followed by a 1200 s dissociation phase. Regeneration was performed by injecting 15 μL of 10 mM glycine, pH 1.5, at 30 μL/min over all FCs. Reference-subtracted data were collected as FC2-FC1, FC3-FC1, and FC4-FC1. The measurements were obtained at 25 °C. The affinity (K_D_) was calculated using either steady-state equilibrium analysis with the Scrubber 2 Biacore Evaluation Software or a “1:1 (Langmuir) binding” model in BIA Evaluation. For FcγRIIa, FcγRIIb, and FcγRIIIa binding, antibodies were diluted to 5 μg/mL in running buffer, and approximately 500 RU of each variant was captured in FCs 2 through 4 (RU captured). FC1 was again the reference FC. Fcγ receptor ECDs were diluted to 10 μM in running buffer and then 2-fold serially diluted in running buffer to 39 nM. Duplicate injections of each concentration were injected over all FCs at 40 μL/min for 60 s followed by a 120 s dissociation phase. Regeneration was performed by injecting 15 μL of 10 mM glycine, pH 1.5, at 30 μL/min over all FCs. Reference-subtracted data were collected as FC2-FC1, FC3-FC1, and FC4-FC1. The measurements were obtained at 25 °C. The affinity (K_D_) was calculated using the steady-state equilibrium analysis with the Scrubber 2 Biacore Evaluation Software.

#### 2.1.5. C1q ELISA

A 96-well microplate was coated with 100 μL/well of each antibody diluted in DPBS (Dulbecco’s HyClone) with a concentration range of 10 μg/mL to 0.19 μg/mL. Testing was performed in duplicate wells. The plate was sealed and incubated overnight at 4 °C. The coating reagent was removed from each well, and 200 μL/well of casein blocking reagent (Thermo) was added. The plate was sealed and incubated for 2 h at room temperature (RT). Each well was washed 3 times with wash buffer (1 × TBE with 0.05% Tween 20). Next, 100 μL/well of human C1q (MP Biomedical, Santa Ana, CA, USA) at 10 μg/mL diluted in casein blocking reagent was added and incubated for 3 h at RT. The plate was then washed three times with wash buffer before 100 μL/well of a 1:800 times dilution of Sheep anti-human C1q-HRP (Abcam #ab46191) in casein blocker was added and incubated for 1 h at RT. The plate was washed 6 times with wash buffer, and 100 μL/well of TMB Substrate (Pierce) was added to each well and incubated for 7 min. Following this, 100 μL of 1 N HCl was added to each well to stop the reaction. Optical density was immediately measured using a colorimetric microplate reader set to 450 nm.

### 2.2. In Vitro Cell-Based Assays

#### 2.2.1. Dilution of Test Samples

The test samples (bamlanivimab, LSN3832595, and LSN3340566) were first diluted to a concentration of 30 μg/mL in the assay medium. The test samples at 30 μg/mL starting concentrations were then aliquoted in triplicate in the first row of the 96-deep well complement-dependent cytotoxicity (CDC) dilution plate (Axygen, P-DW-20-CS, Corning, NY, USA) that was then 4-fold serially diluted. A further 10-fold dilution was performed of the original 30 μg/mL for antibody-dependent cellular cytotoxicity (ADCC) dilution plates resulting in a 3 μg/mL concentration. Four-fold serial dilutions were performed for ADCC dilution plates as described for CDC dilution plates. Once the serial dilutions were completed, a volume of 50 μL of these samples was dispensed from each of the wells of ADCC dilution and CDC dilution plates into the corresponding wells of 96-well CDC and ADCC assay plates, respectively. The assay medium consisted of RPMI1640 (no phenol red) with 0.1 mM non-essential amino acids (NEAA), 1 mM sodium pyruvate, 2 mM L-glutamine, 500 U/mL of penicillin-streptomycin, and 0.1% *w*/*v* BSA.

#### 2.2.2. Preparation of CHO-Spike/hCD20 Target Cells

CHO cell lines overexpressing Spike protein and human CD20 were cultured in LM7312 media with 10 μg/mL Puromycin (CHO-Spike/hCD20 medium). On the day of the experiments, the cells were collected, counted using a Vi-cell Counter, centrifuged at 400× *g* for 5 min and the cells were resuspended in assay medium to a final cell density of 1.0 × 10^6^ cells/mL. A volume of 50 μL cells/well was added to both ADCC and CDC assay plates that had received 50 μL/well of the serially diluted test samples. The contents of the plate were mixed by gentle agitation on a plate shaker for 30 s at 200 rpm. The plates were then incubated for 1 h at 37 °C.

#### 2.2.3. Preparation of Jurkat FcγRIIIa (V158)-NFAT-Luc Cells

Jurkat FcγRIIIa (V158)-NFAT-Luc cell lines stably co-expressing human FcγRIIIa (V158), human FcεRγ-chain and NFAT luciferase reporter gene were cultured in 10% FBS in RPMI1640 containing 1 mM sodium pyruvate, 0.1 mM NEAA, 2 mM L-glutamine and 500 U/mL of penicillin-streptomycin, 0.2 mg/mL hygromycin B, 0.5 mg/mL geneticin, and 200 ng/mL puromycin. On the day of the experiments, the cells were collected in a 50 mL Falcon tube and counted using a Vi-cell Counter. Cells were centrifuged at 400× *g* for 5 min, the growth media were discarded, and the cells were resuspended in assay media to a final cell density of 3 × 10^6^ cells/mL. A volume of 50 μL was added to the ADCC assay plates already containing the serially diluted test samples and CHO-Spike/hCD20 cells. The contents of the plate were mixed by gentle agitation on a plate shaker for 30 s at 200 rpm. The plates were then incubated for 4 h at 37 °C.

#### 2.2.4. Preparation of Complement

Five milliliters of complement from human serum (Quidel, San Diego, CA, USA, A113) was rapidly thawed at 37 °C and six-fold diluted using assay buffer. A volume of 50 μL/well of diluted complement was added to the CDC assay plates already containing the serially diluted test samples and CHO-Spike/hCD20 cells. The plates were then incubated for 2 h at 37 °C.

#### 2.2.5. Luminescence Readout

At the end of the incubation time, the assay plates were brought to RT for 10 min followed by addition of 100 μL of One-glo Ex (E8130, Promega) to the ADCC assay plates and the Cell-Titer Glo (G7571, Promega, Madison, WI, USA) to the CDC assay plates. The contents of the plates were mixed by gentle agitation on a plate shaker for 1 min at 100 rpm. This was followed by incubation at RT for 10 min. The luminescence was read using a BioTek plate reader (BioTek Instruments, Winooski, VT, USA) with a per-well read time of 0.2 s. The results were analyzed using Prism v8.3.1 (Graph pad, Boston, MA, USA).

### 2.3. ADCP Assay

#### 2.3.1. Differentiation of Macrophages from Primary Monocytes

CD14+ cells were freshly isolated from whole blood of three donors using an EasySep Direct Human Monocyte Isolation Kit (StemCell Technologies, Cat#19669, Vancouver, BC, Canada). Cells were resuspended in ImmunoCult-SF Macrophage Medium (StemCell Technologies, Cat# 10961) supplemented with 50 ng/mL M-CSF (PeproTech, Cat# AF-300-25, Rocky Hill, NJ, USA), and plated at 1 × 10^6^ cells/mL in 10 cm cell tissue culture dishes. Monocytes were differentiated into macrophages (phagocytes) over 7 days, with fresh M-CSF (50 ng/mL) added on day 4.

#### 2.3.2. Cell Labeling, Antibody Treatment and Co-Culture, and Data Acquisition

Monocyte-derived macrophages (effector cells) were removed from cell culture dishes by incubating cells with Accutase (ThermoFisher Scientific, Cat#00-4555-56, Waltham, MA, USA). Both effector cells and SARS-CoV-2 Spike-CHO (target cells) were washed with PBS. Cells were incubated in PBS supplemented with either CellTrace CFSE dye (for target cells, ThermoFisher Scientific, Cat#C34554) or CellTrace Violet dye (for effector cells, ThermoFisher Scientific, Cat#C34557), respectively, for 15 min at 37 °C, 5% CO_2_. After three washes in assay media (RPMI 1640 + 10% FBS + 1% P/S/G + NEAA + NaPyr + HEPES + 10 μM BME), CFSE-labeled target cells were resuspended in assay media and 1 × 10^5^ cells/well were added to 96-well U-bottom plates, followed by the addition of anti-SARS-CoV-2 antibodies or IgG1 isotype controls (final concentrations ranging from 6.1 pM to 100 nM). Target cells and antibodies were incubated 15 min at 4 °C. Target cells without antibody were included as a control for background phagocytosis. Effector cells without target cells or antibody were included as a background control. Violet-labeled macrophages (effector cells) were resuspended in the above assay media and were added to the mixture of target cells, with or without titrated antibodies, at 1 × 10^5^ cells/well. Cells were mixed by pipetting and plates were incubated at 37 °C, 5% CO_2_ overnight. Unattached cells were transferred to new plates and pelleted by centrifugation at 500× *g* for 5 min. Supernatants were discarded. Cells in the original plates were detached with Accutase and transferred to the corresponding wells of the new plates containing unattached cells. Cells were washed with AutoMACS running buffer (Miltenyi Biotec (Bergischgrad, Germany) 130-091-221, containing 0.5% BSA, 2 mM EDTA, and 0.09% sodium azide in PBS pH 7.2) once. Cells were resuspended in AutoMACS running buffer and 2 µL of 7-AAD (BD Pharmingen 559925, lot 9134970) was added for live/dead cell staining. After three washes with AutoMACS running buffer, cells were resuspended with the same buffer. All data were acquired using an LSRFortessa X-20 equipped with five lasers (BD Biosciences, Franklin Lakes, NJ, USA). A total of 30,000 events were acquired for each sample.

#### 2.3.3. ADCP Assay Data Analysis

Data were analyzed using FCS Express 7 software (DeNovo Software, Pasadena, CA, USA). Dead cells and cell debris were excluded from the analysis. The Alexa Fluor 488 channel was used to identify target cells (CFSE labeled). The BV421 channel was used to identify the phagocyte population (Violet-labeled). Phagocytosis was quantified by identifying Violet and CFSE double positive cells within the Violet-positive cell population which contained target cells within them (% CFSE and Violet double-positive events in Violet-positive events, the signal readout is shown as X in the following formula). Percent induction of phagocytosis was calculated as follow: 100 × (X − Min)/(Max − Min). Min is the average of signal readouts from no antibody treatment samples. Max is the signal readout from a 100 nM bamlanivimab-treated sample. For EC50 determinations, % induction of ADCP was plotted with GraphPad Prism using a 4-parameter logistic equation over an 8-point dose response curve. An EC50 with a wide range of top values or a top value under 50 is not meaningful for comparison. The experiment was performed across three donors.

### 2.4. In Vivo Methods

#### 2.4.1. Virus

The challenge virus (SARS-CoV-2/INMI1-Isolate/2020/Italy) in this study was isolated from the sputum of the first clinical case in Italy and is certified mycoplasma-free (provided by European Virus Archive goes Global (EVAg); 008 V-03893). As previously described [34], the complete sequence is available on the GISAID website (BetaCoV/Italy/INMI1-isl/2020: EPI_ISL_410545). For in vivo challenge, the isolate was propagated on Vero E6 cells (CRL-1586 ATCC) and the supernatant was collected and clarified by centrifugation (P3 virus stock).

#### 2.4.2. Animal Challenge

SARS-CoV-2 seronegative AGMs (*Chlorocebus aethiops*) (12 males, 11 females, 3.68 to 7.44 kg) (St Kitts origin, PreLabs, Inc., Kelowna, BC, Canada) were randomly assigned to four treatment groups. Investigators that assessed, measured, or quantified the results, as well as animal husbandry staff, were blinded to the treatment group. For experimental infection, AGMs were anesthetized with ketamine and inoculated with 8.0 × 10^5^ PFU of SARS-CoV-2 (SARS-CoV-2/INMI1-Isolate/2020/Italy) via combined i.n. and i.t. routes (dose divided evenly; 1.0 mL total, 0.5 mL per nostril). Animals were monitored for signs of illness and scored daily for appetite, activity level, posture, and respiration rate according to pre-established criteria (scale of 0–20); a clinical score ≥ 9 warrants euthanasia. The 5 DPI data collection end point was predetermined based on previous experiments, as this time point correlates with peak viral shedding and pathology. Animals were sedated using ketamine or telazol for all measurements requiring physical manipulation. The animal protocol was approved by the University of Texas Medical Branch (UTMB) Institutional Animal Care and Use Committee and adheres to the NIH Guide for the Care and Use of Laboratory Animals.

#### 2.4.3. Virus Titration

Infectious SARS-CoV-2 viral loads and virus stocks were titrated by plaque assay on Vero E6 cells. Briefly, increasing ten-fold dilutions of samples or stocks were adsorbed to monolayers in duplicate or quadruplicate. After an hour of incubation, cells were overlaid with EMEM agar medium plus 1.25% Avicel and incubated for two days at 37 °C in 5% CO_2_. Plaques were counted after staining plates with 1% crystal violet in formalin.

#### 2.4.4. RNA Isolation from SARS-CoV-2-Infected AGM Samples

On specified procedure days, 100 μL of blood, neat BAL fluid, or mucosal swab (nasal, oral, rectal) samples from each animal was added to 600 μL of AVL viral lysis buffer (Qiagen). Tissues were put in RNAlater and inactivated with Qiagen RLT buffer. Following removal from the high-containment laboratory, RNA was isolated from blood, BAL, and swabs using the QIAamp viral RNA kit (Qiagen). Tissue RNA was extracted using a Qiagen RNeasy Mini kit.

#### 2.4.5. Detection of SARS-CoV-2 Load from AGMs

One-Step Probe RT-qPCR kits (Qiagen) and CFX96 system/software (BioRad, Hercules, CA, USA) were used to determine the concentration of viral copies in samples. To detect SARS-CoV-2 RNA, we targeted the nucleoprotein (N) using CDC SARS-CoV-2 N2 assay primer/probe sets.Thermocycler run settings were 50 °C for 10 min, 95 °C for 10 s, and 45 cycles of 95 °C for 10 s and 55 °C for 30 s. Threshold cycle (*C_T_*) values representing SARS-CoV-2 genomes were analyzed with CFX Manager Software, and data are presented as genome equivalents (GEq). To generate the GEq standard curve, SARS-CoV-2 RNA from cell supernatants was serially diluted, and the number of genomes was calculated using Avogadro’s number and the molecular weight of the SARS-CoV-2 genome.

#### 2.4.6. Hematology and Serum Biochemistry

White blood cell differentials and platelet counts in AGM whole blood were monitored with a laser-based Vetscan HM5 hematologic analyzer (Abaxis, Union City, CA, USA). Serum concentrations of liver enzymes (alanine aminotransferase (ALT), aspartate aminotransferase (AST), alkaline phosphatase (ALP), gamma-glutamyl transferase (GGT)); kidney products (blood urea nitrogen, creatinine); C-reactive protein; glucose; and amylase were measured with a Piccolo point-of-care analyzer and Biochemistry Panel Plus discs (Abaxis). Coagulation testing was accomplished with a VETSCAN VSpro (Abaxis) and equine fibrinogen and canine PT/aPTT combination cartridges.

#### 2.4.7. Serum ELISAs for Human IgG Concentrations

Concentrations of human IgG in AGM serum were determined by an ELISA assay. Goat anti-Human Kappa Monkey ads-UNLB (Southern Biotech, Birmingham, AL, USA, Catalog Number 2064-01; 1.00 μg/mL) was coated on the ELISA plate (ThermoFisher Scientific, Catalog Number 3855 or equivalent) as the capture reagent. Calibrators, controls and samples in neat rhesus macaque serum were diluted 10–40-fold and transferred to the coated plates. After incubation, the plate was washed to remove unbound material, and mouse anti-human IgG Fc-HRP (Southern Biotech, Catalog Number 9040-05; 10 ng/mL) was added as the detection reagent. Following incubation, unbound enzyme was washed away and BioFX^®®^ TMB One Component HRP Microwell Substrate (SurModics, Eden Prairie, MN, USA, Catalog Number TMBW-0100-01 or equivalent) was added to the wells. Color development was stopped by the addition of Phosphoric Acid (Fisher Chemical, 19 Catalog Number A260-500 or equivalent) and the optical density was measured at 450 nm with wavelength correction set to 650 nm. Immunoreactivity was determined from calibrators using a 4-parameter logistic (Marquardt, Rithem-Weilheim, Germany) regression model with 1/F2 weighting (Watson Bioanalytical LIMS, Version 7.4.2 SP1).

#### 2.4.8. Serum Neutralization Assay

Antibody neutralization titers were calculated by determining the dilution of serum that reduced 50% of viral plaques (PRNT_50_). A standard 100 PFU amount of SARS-CoV-2 was incubated with two-fold serial dilutions of serum samples for one hour. The virus–serum mixture was then inoculated onto Vero E6 cells for 60 min and cells were overlaid with EMEM agar medium plus 1.25% Avicel. After a 48 h incubation, plates were stained with 1% crystal violet in formalin and plaques counted.

#### 2.4.9. Bead-Based Cytokine and Coagulation Immunoassays

Concentrations of circulating immune mediators were determined by flow cytometry using LegendPlex multiplex technology (BioLegend, San Diego, CA, USA). Serum levels of cytokines/chemokines were quantified using Non-human Primate Inflammation 13-plex (1:4 dilution) and Human Thrombosis (1:100 dilution) kits (BioLegend). Samples were processed in duplicate following the kit recommendations. Following bead staining and wash steps, 3000–4000 bead events were acquired using BD FACS Diva software (Version 8.0) and a FACS Canto II cytometer (BD Biosciences). The raw files were analyzed with cloud-based LEGENDplex™ Data Analysis Software (Version 5) (BioLegend).

### 2.5. Pathology and Immunohistochemistry

#### 2.5.1. Pathology

Necropsy was performed on all animals at euthanasia or the study end point (5 DPI). Lung lesions were interpreted by a board-certified veterinary pathologist and scored according to the following criteria: 0 (0% of lobe affected), 1 (1% to 25% of lobe affected), 2 (26% to 50% of lobe affected), 3 (51% to 75% of lobe affected), 4 (76% to 100% of lobe affected). The following formula was used to calculate the mean gross score: [(right upper lobe + right median lobe + left upper lobe + left median lobe)/4 + (right lower lobe + left lower lobe)/2]/2.

#### 2.5.2. Immunohistochemistry

The lungs were collected for histopathologic and immunohistochemical (IHC) examination, as described previously [34]. Samples were immersion-fixed in 10% neutral buffered formalin for a period of >7 days. Processed samples were embedded in paraffin and sectioned at a thickness of 5 μm. For IHC, specific anti-SARS immunoreactivity was detected using an anti-SARS nucleocapsid protein rabbit primary antibody at a 1:800 dilution for 1 h (Novusbio, Centennial, CO, USA). The tissue sections were processed for IHC with a ThermoFisher Scientific Lab Vision Autostainer 360 (ThermoFisher Scientific). Sections were incubated with a biotinylated goat anti-rabbit IgG secondary antibody (Vector Laboratories, Burlingame, CA, USA) at a 1:200 dilution for 30 min followed by Vector Streptavidin Alkaline Phosphatase at a dilution of 1:200 for 20 min (Vector Laboratories, Burlingame, CA, USA). Slides were developed with Bio-Red (Biopath Laboratories, Oklahoma City, OK, USA) for 7 min and counterstained with hematoxylin for 60 s. Fibrin deposition was detected using an anti-fibrin monoclonal mouse primary antibody at a 1:3200 dilution for 60 min (Sekisui Diagnostics, Burlington, MA, USA) and processed with an autostainer. Sections were incubated with biotinylated goat anti-mouse IgG secondary antibody (Vector Laboratories, Burlingame, CA, USA) at a dilution of 1:200 for 30 min followed by Vector Streptavidin Alkaline Phosphatase at a dilution of 1:200 for 20 min (Vector Laboratories, Burlingame, CA, USA). Again, slides were developed with Bio-Red (Biopath Laboratories, Oklahoma City, OK, USA) for 7 min and counterstained with hematoxylin for 60 s.

#### 2.5.3. Pancytokeratin

Tissue sections were deparaffinized and rehydrated through xylene and graded ethanols. Slides went through heat antigen retrieval in a steamer at 95 °C for 20 min in Sigma Citrate Buffer, pH 6.0, 10× (Sigma Aldrich, St. Louis, MO, USA). To block endogenous peroxidase activity, slides were treated with a 3% hydrogen peroxide and rinsed in distilled water. The tissue sections were processed for IHC using the Thermo Autostainer 360 (ThermoFisher, Kalamazoo, MI, USA). Sequential 15 min incubations with avidin D and biotin solutions (Vector Laboratories, Burlingame, CA, USA, #SP-2001) were performed to block endogenous biotin reactivity. Specific anti-pan Cytokeratin (AE1/AE3) was detected using an anti-pan Cytokeratin (AE1/AE3) primary antibody at a 1:80 dilution for 60 min. Secondary antibody used was biotinylated goat anti-mouse IgG (Vector Laboratories, Burlingame, CA, USA, #BA-9200) at 1:200 for 30 min, followed by Vector Horseradish Peroxidase Streptavidin, R.T.U. (Vector Laboratories #SA-5704) for 30 min. Slides were developed with Dako DAB chromogen (Dako, Carpenteria, CA, USA, #K3468) for 5 min and counterstained with hematoxylin for 45 s.

### 2.6. Statistics

#### Virology Statistics

Data were analyzed using GraphPad Prism 9.3.1 software and R [35]. Multiple imputation (m = 20 imputations) was conducted in accordance with standard procedures described in [36]. All statistical analyses were performed using log10 transformed viral response values. Imputation of left-censored data was performed using random normal values from a distribution having mean log10 (LOD) minus 2.326 s, where s was estimated by the residual error term (RMSE) from a linear model fit of all non-censored data. Following imputation, a standard MMRM (mixed model repeated measures) model was fitted using animal as a random effect, group, day, and group*day as fixed effects, and an unstructured covariance matrix. Effects were pooled in accordance to [36] to estimate a pooled effect size, standard error, and *p*-value. Pooled *p*-values were estimated from a t-distribution with the degrees of freedom derived from the method described by Barnard and Rubin [37]. Within study pooled *p*-values were then adjusted for multiplicity using the Benjamini–Hochberg method. Reported statistics are *p*-values and *q*-values, with *q* < 0.05 indicating statistical significance.

## 3. Results

### 3.1. Evaluation of In Vitro ADE

FcγR expression was analyzed by flow cytometry of immortal and primary cells used in these experiments. FcγRIa and FcγRIIa were detected on THP-1 cells, FcγRIIa and FcγRIIb were detected on Raji cells and FcγRI, FcγRIIa, FcγRIIb, and FcγRIII were all detected on primary human macrophages. None of the FcγRs examined were detected on ST486 cells (Figure S1a,b). Our panel of cells—monocytic THP-1, B-lymphocytic Raji, and primary macrophages—expressed a relevant repertoire of Fcγ receptors to inform the risk of FcR-mediated ADE. Further, ST486 cells were used as a negative control, whereas the SARS-CoV-2 permissive cell line, Vero-E6 (derived from AGM), was used as the positive control.

Each of the cell types was incubated at a multiplicity of infection of 0.1 with a clinical isolate of SARS-CoV-2 in the presence of varying concentrations of either bamlanivimab (0.3–3000 ng/mL) or a nonspecific IgG1 isotype control antibody to assess ADE of SARS-CoV-2 infection. In Vero-E6 cells (positive control), intracellular and extracellular viral RNA were quantifiably detected at 6 and 48 h post infection (hpi) (Figure S2a–l). At 6 hpi, treatment with bamlanivimab reduced both positive- and negative-strand intracellular RNA levels in a dose-dependent manner compared with samples treated with an IgG1 isotype control (Figure S2a,b). Extracellular RNA levels were below the lower limit of quantification (LLOQ) at 6 hpi (Figure S2c). At 48 hpi, only samples treated with the highest dose of bamlanivimab (3000 ng/mL) exhibited a decrease in viral RNA levels compared with samples treated with IgG1 isotype control (Figure S2d–f). Viral RNA was below the LLOQ at both time points under all conditions in ST486 cells (negative control) (Figure S2g–l).

**Figure 1:**
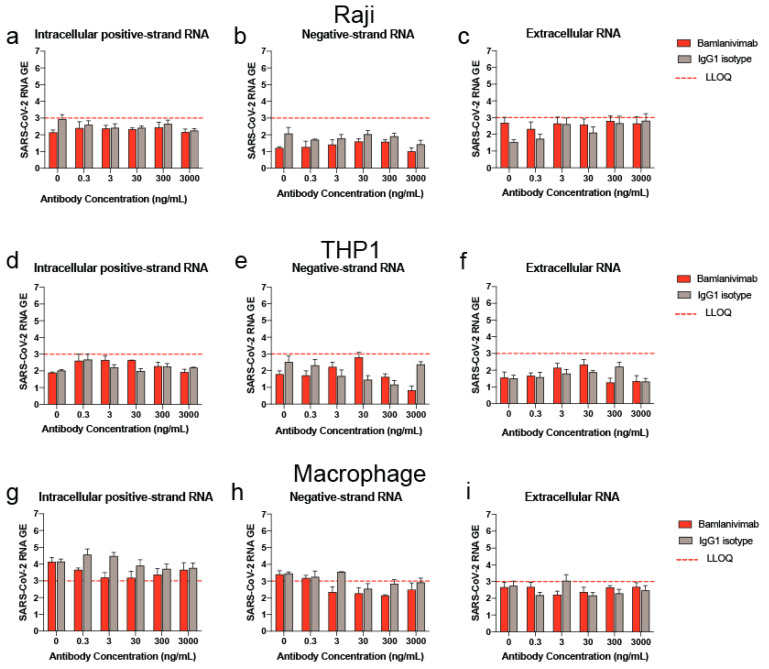
**Quantification of viral RNA in Raji, THP1 and primary human macrophage at 48 h post-infection (hpi).** Intracellular positive-strand RNA, intracellular negative-strand RNA and extracellular viral RNA in Raji cells (**a**–**c**), THP1 cells (**d**–**f**) and representative primary human macrophages (**g**–**i**) at 48 hpi with SARS-CoV-2 at 0.1 MOI in the presence or absence of bamlanivimab (0.3–3000 ng/mL) or IgG1 isotype control (0.3–3000 ng/mL). Data from one experiment (out of two experiments) is shown here. Bars denote mean +/− SD (N = 3 per sample). GE, genome equivalents (LOG10) of SARS-CoV-2 in cells at 48 hpi with the virus at an MOI of 0.1.

Bamlanivimab did not increase viral RNA production at any concentration in FcγR-expressing cell lines. At 48 hpi, with bamlanivimab or IgG1 isotype control, intracellular and extracellular viral RNA levels were below the LLOQ in both Raji and THP-1 cells for all treatment groups (control, bamlanivimab 0.3–3000 ng/mL and IgG1 0.3–3000 ng/mL), (Figure 1a–f). In primary human macrophages from two donors, intracellular and extracellular viral RNA levels also were below the LLOQ in macrophages for all treatment groups (data not shown). In macrophages from a third donor, intracellular positive-strand RNA was quantifiably detected in all treatment groups at 48 hpi. However, no increases in viral RNA were observed with bamlanivimab compared to control (Figure 1g–i). Bamlanivimab also did not significantly increase viral RNA levels in any of these cell types at 6 hpi (Figure S3a–i).

### 3.2. FcγR Binding

The potential for bamlanivimab to mediate effector functions was assessed by measuring its binding to FcγRs. The affinity (K_D_) of bamlanivimab to the human FcγRI, FcγRIIa, FcγRIIb, and FcγRIIIa receptor extracellular domains (ECDs) was measured using surface plasmon resonance (SPR) (Table S1). Bamlanivimab demonstrated comparable binding to FcγRI, FcγRIIa, FcγRIIb, and FcγIIIa as the human IgG1 positive control antibody, LSN2436595. In contrast, the human IgG4 PAA negative control antibody, LSN2835015, showed weaker binding to the human FcγRI, FcγRIIa, FcγRIIb, and FcγRIIIa receptor ECDs in both SPR experiments as expected.

### 3.3. Antibody-Dependent Cell-Mediated Cytotoxicity and Complement-Dependent Cytotoxicity

**Figure 2:**
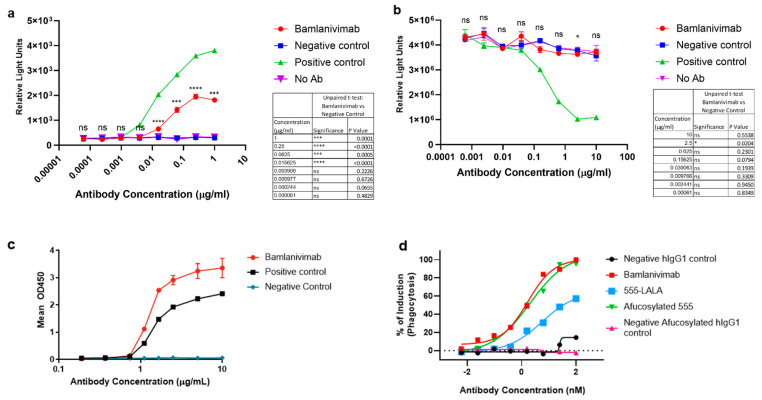
**In vitro effector function.** Cell-based assays to investigate (**a**) antibody-dependent cell-mediated cytotoxicity (ADCC), (**b**,**c**) complement-dependent cytotoxicity (CDC) and (**d**) antibody-dependent cellular phagocytosis (ADCP). (**a**) FcγRIIIa (ADCC) activity mediated by bamlanivimab. LSN3832595 is IgG4-PAA negative isotype control for bamlanivimab and LSN3340566 is an anti-CD20 IgG1 antibody and was used as an alternative positive control for these assays. (**b**) CDC activity mediated by bamlanivimab. These data are representative of three independent assay runs. (**c**) C1q ELISA binding results for bamlanivimab. Optical density (OD) results at 450 nm wavelength (mean ± SD) from 1 of 3 experiments performed are shown. Four parameter logistic curve fitting. (**d**) Flow cytometry analysis of ADCP activity of bamlanivimab, afucosylated 555, and (555-LALA) antibody dose–response curves. Two positive controls, hIgG1 LSN2436595 and afucosylated hIgG1 LSN2436595, were included in the studies. The experiments were performed using three donors. A representative donor is shown.

Next, we investigated the ability of bamlanivimab to trigger in vitro ADCC and CDC upon engagement of the viral spike protein (Spike). Jurkat cells stably co-expressing human FcγRIIIa (V158), human FcεRγ-chain, and luciferase under the control of the NFAT response element were used as ADCC reporter cells. Chinese hamster ovary (CHO) cells stably expressing Spike and human CD20 were used as target cells (CHO-Spike/hCD20). As shown in Figure 2a, bamlanivimab stimulated FcγRIIIa-dependent ADCC activity in reporter cells after engagement of CHO-Spike/hCD20 target cells (Figure 2a). As expected, the anti-spike antibody with IgG4-PAA isotype, used as a negative control, did not stimulate our ADCC assays. The humanized anti-CD20 IgG1 antibody (LSN3340566, positive control) showed robust ADCC and CDC activities (Figure 2a,b). Interestingly, despite demonstrating binding to human C1q by ELISA (Figure 2c), bamlanivimab failed to elicit CDC activity in cell-based assays (Figure 2b).

### 3.4. Antibody-Dependent Cellular Phagocytosis

FcR-mediated effector functions, such as antibody-dependent cellular phagocytosis (ADCP), may be important components of the anti-viral activity of SARS-CoV-2 neutralizing mAbs. To test the ability of bamlanivimab to trigger ADCP activity, CHO cells stably expressing SARS-CoV-2-spike protein were generated as targets, and human monocyte-derived macrophages endogenously expressing FcγRs were used as effector cells. Target cells were labeled with CellTrace CFSE dye and effector cells were labeled with CellTrace Violet dye. Flow cytometry was used to measure the uptake of target cells into macrophages; percent induction of phagocytosis was defined as %Violet and CFSE double-positive cells in Violet-positive cells, normalized to maximum and minimum signals. Bamlanivimab demonstrated dose-dependent ADCP activity when co-cultured with SARS-CoV-2 spike protein expressing CHO-Spike cells (Figure 2d). No difference in ADCP activity was observed between bamlanivimab and an afucosylated version of the molecule (afucosylated 555). In three test donors, the mAb with a LALA mutation in the Fc region (555-LALA) showed a significant reduction in ADCP activity compared with bamlanivimab and afucosylated 555 (Figure 2d). These data demonstrate that bamlanivimab triggers ADCP activity in in vitro cell-based assays and this is contingent on its Fc portion.

### 3.5. In Vivo Study Design

**Figure 3:**
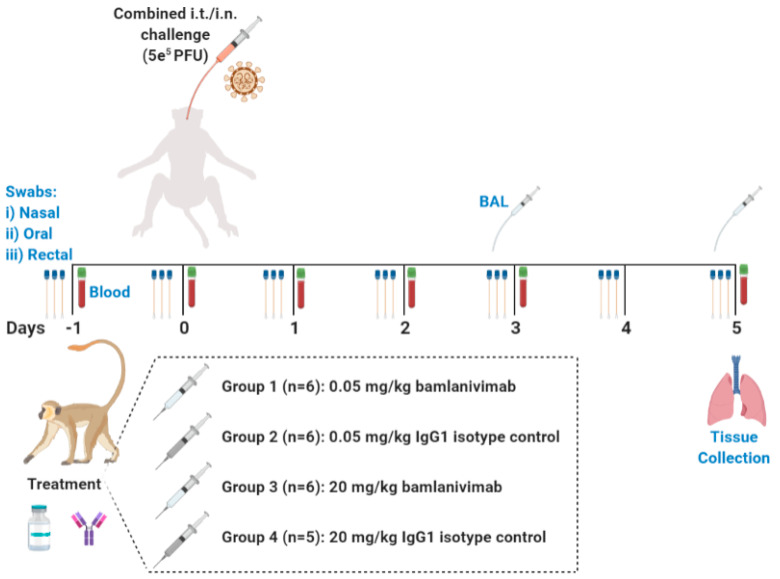
**In vivo study design.** African Green Monkey experimental design.

Twenty-three adult AGMs (12 males and 11 females) were randomly assigned to four treatment groups. The following were intravenously (i.v.) administered: (a) a single 0.05 mg/kg bamlanivimab sub-saturating dose (n = 6), (b) 0.05 mg/kg IgG1 isotype control sub-saturating dose (n = 6), (c) 20 mg/kg bamlanivimab saturating dose (n = 6), or (d) 20 mg/kg IgG1 isotype control saturating dose (n = 5) (Figure 3). Twenty-four hours after treatment, AGMs were exposed to 5 × 10^5^ plaque-forming units (PFU) of SARS-CoV-2 (Italian isolate, INMI 1) via combined intratracheal (i.t.) and intranasal (i.n.) routes (dose divided equally). Animals were scored twice daily for signs of illness. Viral loads were measured in mucosal swabs (nasal, oral, rectal) collected daily and bronchoalveolar lavage (BAL) samples collected at 3 and 5 days post infection (DPI). Blood samples were taken at 0, 1, 2, 3, and 5 DPI to assess changes in hematology, clinical biochemistry, and coagulation parameters, and to monitor drug decay and systemic levels of inflammatory mediators. Based on previous studies, the study end point was predefined as 5 DPI, which represents the peak stage of disease and virus shedding in AGMs. To evaluate treatment efficacy, bamlanivimab and isotype control-treated cohorts were compared at equivalent doses (0.05 mg/kg or 20 mg/kg) to determine dose-dependent effects.

After challenge, a single animal that received a sub-saturating dose of bamlanivimab experienced severe respiratory distress, prolonged recumbency, and anorexia necessitating euthanasia at 4 DPI. All other animals treated with varying doses of isotype control or bamlanivimab survived to study termination at 5 DPI. All cohorts showed evidence of decreased appetite at 4 and 5 DPI, possibly attributable to the cumulative effect of repeated anesthesia. No significant differences were found between the experimental groups in terms of weight, temperature, or biscuit intake (data not shown).

### 3.6. Quantification of SARS-CoV-2 Viral Loads in AGMs

To assess whether pretreatment with bamlanivimab resulted in ADE of SARS-CoV-2 infection, viral loads were monitored by reverse transcriptase-polymerase chain reaction (RT-qPCR) and infectious viral loads were quantified by plaque assay on Vero-E6 cells. Most importantly, there was no evidence of increased viral loads in any of the bamlanivimab treatment groups relative to isotype control-treated groups. All animals had observable quantities of viral RNA in nasal and oral secretions that peaked at 2 DPI in isotype control animals. Neither viral RNA nor infectious virus titers were discernible by RT-qPCR in whole blood or plasma of animals, indicating a lack of peripheral circulation of cell-associated or free virus (data not shown).

**Figure 4:**
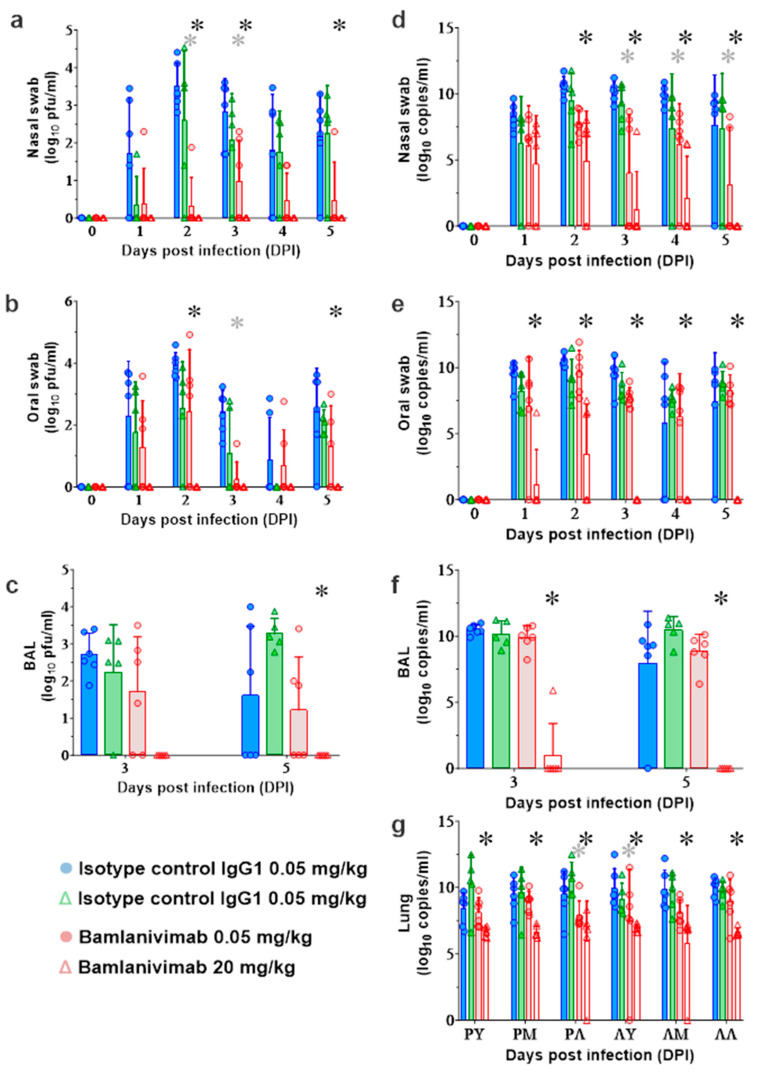
**In vivo virology data.** AGM viral loads in mucosal swabs and BAL samples. Viral loads as titrated by (**a**–**c**) plaque assay or (**d**–**g**) RT-qPCR. Nasal swabs (**a**,**d**), oral swabs (**b**,**e**), BAL fluid (**c**,**f**) and lung tissue samples (**g**) were collected from AGMs infected with SARS-CoV-2 and treated with a bamlanivimab sub-saturating dose (n = 6), IgG1 isotype control sub-saturating dose (0.05 mg/kg) (n = 6), bamlanivimab saturating dose (n = 6), or IgG1 isotype control saturating dose (n = 5) (20 mg/kg). Bars show the mean of duplicate RT-qPCR reactions or duplicate wells and lines represent standard deviation. Data below lower level of quantification (LLOQ) included as LLOQ/2. RT-PCR LLOQ is 10,000 copies/mL. Plaque assay LLOQ was 25 PFU/mL. DPI, days post infection; BAL, bronchoalveolar lavage; RU, RM, and RL, right upper, middle, and lower lung. LU, LM, and LL, left upper, middle, and lower lung. Gray star denotes q value < 0.05 that represent statistically significant differences between 0.05 mg/kg bamlanivimab- and isotype control-treated animals. Black star denotes q value < 0.05 that represent statistically significant differences between 20 mg/kg bamlanivimab- and isotype control-treated animals.

Treatment with bamlanivimab reduced viral loads in nasal and oral swabs and BAL fluid (Figure 4a–c), relative to control groups. In the sub-saturating isotype control group, viral loads in nasal and oral swabs differed by approximately 2 to 4 logs, compared with less than 0.5 to 2 logs in the sub-saturating bamlanivimab group (Figure 4a,b), which also led to minimal log reductions of viral loads in BAL fluid (Figure 4c). At 2 DPI, viral RNA titers in isotype control animals ranged between approximately 1 × 10^9^ to 1 × 10^10^ copies per mL (Figure 4d) compared to approximately 1 × 10^7^ and 1 × 10^9^ copies per mL in animals treated with a low dose of bamlanivimab (Figure 4e). At 5 DPI, levels of viral RNA in animals treated with a saturating dose of bamlanivimab were below the assay threshold in nasal swabs and BAL fluid and at 3 to 5 DPI in oral swabs (Figure 4d–f). Additionally, treatment with a 20 mg/kg dose of bamlanivimab decreased viral replication in lung tissue, with an average titer reduction of ~3–4 logs of viral genomes in each lobe compared to isotype control animals (Figure 4g).

### 3.7. Clinical Pathology and Quantification of Serum Inflammatory Mediators

An acute phase response consists of mild-to-moderate increases in fibrinogen and C-reactive protein, or minimal-to-mild decreases in albumin, or all three. Acute phase responses were observed as early as day-1 (pretreatment) and persisted in all groups following inoculation with SARS-CoV-2. For males administered 0.05 and 20 mg/kg, there was a bamlanivimab-related reduction in this acute phase response as early as 1 DPI. For males administered 20 mg/kg, this reduction included decreases in C-reactive protein and increases in albumin, along with decreases in fibrinogen. For males at 0.05 mg/kg, the reduced acute phase response was limited to increases in albumin when compared with the respective IgG1 isotype control. Clear alterations in clinical pathology parameters were not observed in female monkeys. The reduction in the acute phase response (data not shown) correlated with microscopic attenuation of mixed cell inflammation of bronchi-bronchioles for males at 20 mg/kg.

Increased serum levels of inflammatory mediators were found in control animals. Minimal changes were evident in bamlanivimab-treated AGMs; however, due to variability, the changes could not be definitively related to treatment (Figure S4). Specifically, IL-6, IP-10, and monocyte chemoattractant protein were elevated at different times throughout the study in isotype control animals. Notably, IL-6 is related to respiratory distress in human COVID-19 cases [38] and was almost 600-fold higher in the single fatal AGM.

### 3.8. Histology and Immunohistochemistry

One AGM in the 0.05 mg/kg bamlanivimab group was humanely euthanized on day 4 (one day prior to scheduled sacrifice) due to clinical signs characterized by abnormal respiration (shallow, sporadic breathing), lateral recumbency, decreased food consumption, and decreased body temperature. Moderate broncho-interstitial pneumonia, similar to the findings in the surviving animals, was microscopically observed. Gross examination of all animals at terminal necropsy revealed varying degrees of red discoloration with or without pulmonary consolidation in all monkeys that correlated with microscopic changes consistent with viral pneumonia. Several AGMs also presented with hemorrhagic pleural effusion.

**Figure 5:**
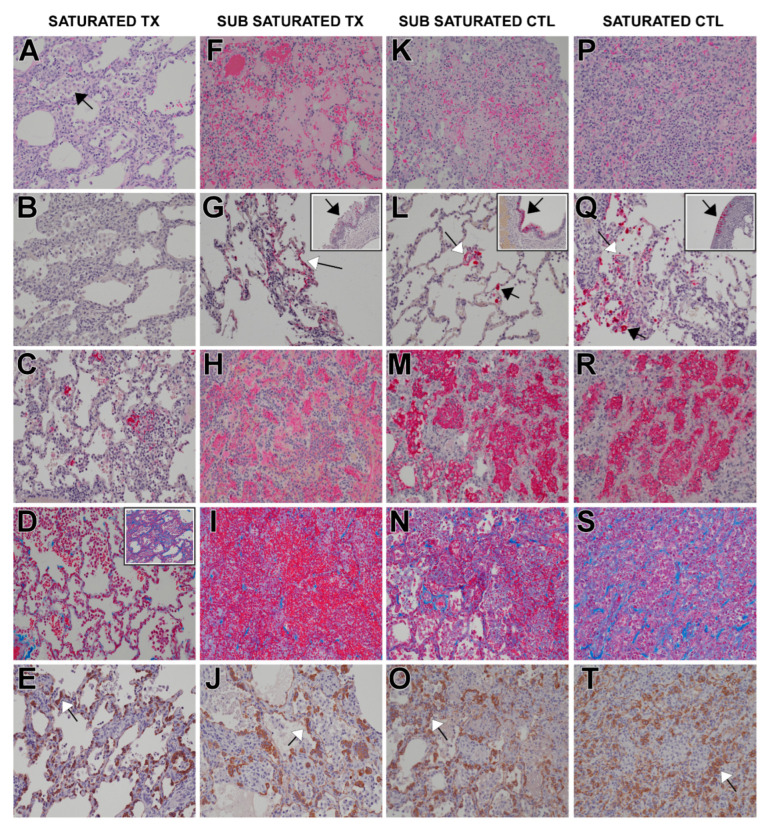
**In vivo lung immunohistochemistry.** (**A**–**E**) Representative sections from saturated antibody dose: (**A**) interstitial pneumonia with increased macrophages (black arrow); (**B**) negative SARS-CoV-2 labeling; (**C**) fibrin accumulation within alveoli (red); (**D**) (inset) collagen deposition (blue) in septum; (**E**) type II pneumonocyte hyperplasia (white arrow) highlighted with pancytokeratin IHC (brown). (**F**–**J**) Representative sections from sub-saturated antibody dose group: (**F**) interstitial pneumonia, alveolar edema, hemorrhage, mixed inflammation with neutrophils, macrophages, and lymphocytes; (**G**) SARS-CoV-2 IHC labeled (red) type I pneumocytes (white arrows) and bronchial respiratory epithelium (inset, black arrow); (**H**) alveoli with fibrin (red); (**I**) collagen deposition (blue) in septum; (**J**) type II pneumocyte hyperplasia (white arrow) highlighted with pancytokeratin IHC (brown). (**K**–**O**) Representative sections from sub-saturated antibody dose control: (**K**) interstitial pneumonia with alveolar edema, hemorrhage, mixed inflammation with degenerative neutrophils, macrophages, and lymphocytes; (**L**) SARS-CoV-2 IHC labeled (red) type I (white arrows) and type II (black arrow) pneumocytes and bronchial respiratory epithelium (inset, black arrow); (**M**) alveoli with fibrin (red); (**N**) collagen deposition (blue) in septum; (**O**) type II pneumocyte hyperplasia (white arrow) highlighted with pancytokeratin IHC (brown). (**P**–**T**) Representative sections from saturated antibody dose control: (**P**) interstitial pneumonia with alveolar edema, hemorrhage, mixed inflammation with degenerative neutrophils, macrophages, and lymphocytes; (**Q**) SARS-CoV-2 IHC positive (red) type I (white arrows) and type II (black arrow) pneumocytes and bronchial respiratory epithelium (inset, black arrow); (**R**) alveoli with fibrin (red); (**S**) collagen deposition (blue) in septum. H&E staining 20× (**A**,**F**,**K**,**P**), SARS-CoV2 IHC 20× (red) (**B**,**G**,**G inset**,**L**,**L inset**,**Q**,**Q inset**), fibrin IHC 20× (red) (**C**,**H**,**M**,**R**), Trichrome staining 20× (blue) (**D**,**D inset**,**I**,**N**,**S**), cytokeratin IHC 20× (brown) (**E**,**J**,**O**,**T**).

The findings were consistent with varying severities of broncho-interstitial pneumonia induced by SARS-CoV-2, and with previous publications of experimental SARS-CoV-2 infection in AGMs [31,32,34]. Predominant microscopic findings driving overall severity of inflammation and injury consisted of mixed cell inflammation of bronchi and bronchioles, increased alveolar/septal cellularity, and hemorrhage and congestion (Table 1). These findings were accompanied by other characteristics of inflammation that included bronchiolitis obliterans, alveolar necrosis, type II pneumocyte hyperplasia, and alveolar syncytial cells. Immunohistochemical labeling of lung tissue for viral antigen demonstrated the presence of viral particles in epithelial cells lining bronchi and bronchioles, with lower amounts observed in type I pneumocytes and alveolar macrophages. Viral antigen was not detected in any of the lung tissue from animals treated with the highest dose of bamlanivimab (Figure 5).

**Figure 6:**
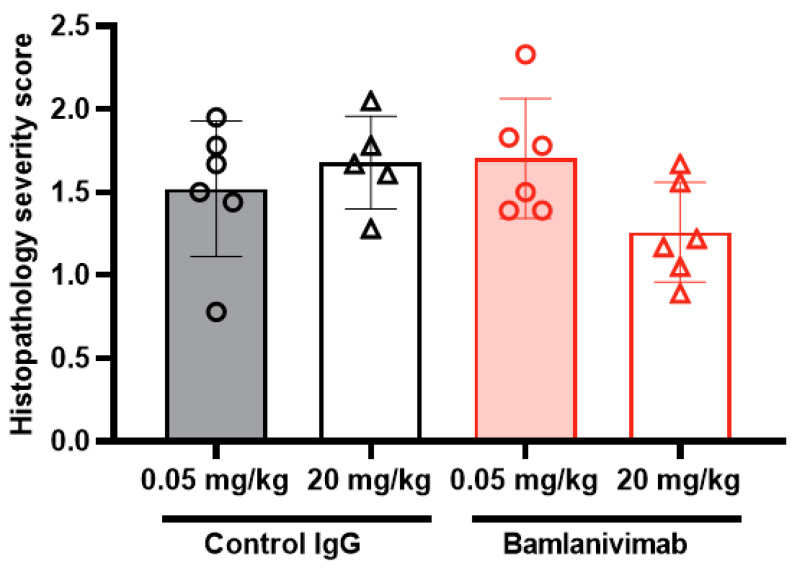
**Individual and group mean severity composite scores.** AGMs infected with SARS-CoV-2 and treated with a 0.05 mg/kg bamlanivimab sub-saturating dose (n = 6), a 0.05 mg/kg IgG1 isotype control sub-saturating dose (n = 6), a 20 mg/kg bamlanivimab saturating dose (n = 6), or a 20 mg/kg IgG1 isotype control saturating dose (n = 5). Data plotted are the composite scores for microscopic findings in AGMs.

To elucidate potential group differences in the severity of microscopic findings, microscopic severity and composite severity scores for the predominant findings were generated for individual animals, which were then used to generate group comparisons (Figure 6). Prominent differences between treatment groups were not observed. Animals treated with 0.05 mg/kg bamlanivimab were most similar to isotype control-treated animals, with the exception of mildly increased congestion and hemorrhage scores, which were largely attributable to the single early-sacrifice animal (Figure 6). Taken together, the microscopic findings along with the decreases in viral loads observed in animals treated with bamlanivimab indicated that ADE of disease was not observed in this study. Mildly decreased severity scores were observed in animals treated with 20 mg/kg of bamlanivimab. Although biologic variation in the presentation of disease could not be excluded, these decreases were compatible with attenuation of SARS-CoV-2 induced disease.

## 4. Discussion

Direct anti-viral mechanisms are central to protection by antibodies while their interactions with FcγRs on immune cells induce downstream mechanisms that further enhance antibody efficacy in vivo [7]. A major concern during the development of safe and effective COVID-19 therapies is the potential enhancement of SARS-CoV-2 infection by ADE, which may occur by several mechanisms, including binding of opsonized viruses to FcγRs to mediate entry into myeloid cells [8,26]. Anti-HIV mAbs and anti-Ebola vaccines have both been shown to activate FcγR pathways leading to protective effects without evidence of ADE [4,7]. The anti-SARS-CoV-2 mAb, bamlanivimab, binds FcγRIIIa, an activating FcγR that promotes ADE in some instances, such as for DENV infection [39]. The data regarding the risk of in vivo ADE of SARS-CoV-2 infection is only now becoming robust [40,41] because early studies suffered from a lack of statistical power [16,17,18,19]. Non-peer-reviewed data suggest that some SARS-CoV-2 neutralizing mAbs can enhance infection by SARS-CoV-2 pseudotypes in vitro [23,42]. However, these findings did not translate into in vivo disease enhancement [42]. More recent reports have described mAbs that do not mediate ADE of SARS-CoV-2 infection in vitro using CHO cells or in vivo using hamster and NHP models [28,29]. Limited data exist for antibodies elicited by COVID-19 vaccines, but there has been little evidence of ADE in vaccinated individuals [43,44]. Since the ability of mAbs and vaccines to exacerbate COVID-19 via ADE in vivo remains unclear, this area warrants continued research to ensure the safety of current and future therapies.

Here, we asked the question of whether bamlanivimab, a COVID-19 mAb, can enhance SARS-CoV-2 infection by ADE both in vitro and in an NHP model of COVID-19. Bamlanivimab possesses in vitro ADCC function, which may be an aspect of its in vivo potency, supported by recent data indicate that engaging activating FcRs results in optimal anti-SARS-CoV-2 antibody protection [45]. Here, the effector function of bamlanivimab was characterized by Fc receptor binding, ADCC, ADCP, complement binding, and CDC assays. Overall, our in vitro data showed that bamlanivimab possesses a potent effector function, as expected for a human IgG1 molecule. Additionally, ADCP analysis suggested that phagocytosis activity and afucosylation of bamlanivimab does not impact ADCP activity. Importantly, bamlanivimab did not enhance the SARS-CoV-2 infection of Fc receptor-expressing cell lines or primary human macrophages. The potential for ADE of infection mediated by bamlanivimab was further explored in an NHP model, which demonstrates homologous clinical and histopathological signs and symptoms of human SARS-CoV-2 infection. Enhanced infection and disease progression were not observed in the presence of bamlanivimab in vivo. These findings are consistent with data for other authorized anti-SARS-CoV-2 mAbs and convalescent plasma [32,34].

First, we investigated the potential for bamlanivimab to mediate SARS-CoV-2 ADE in cells in vitro. Previous studies with the SARS-CoV-1 and the Middle East respiratory syndrome (MERS) coronaviruses indicated that neutralizing mAbs could increase the uptake of virus or pseudovirus comprising spike proteins into FcR-expressing cells [16,18]. To account for Fc receptor-dependent ADE, we used cells that do not express the SARS-CoV-2 canonical ACE2 receptor, but do express FcγRs including, Raji, THP1 cells, and primary human macrophage. In all cases, in the presence of bamlanivimab concentrations ranging from the IC50 to more than 100-fold above IC50, there was no observed increase in cell-associated or secreted viral RNA. This is in line with previous studies that suggest that SARS-like coronaviruses do not replicate in leukocytes [7]. These findings, however, are in contrast to an authorized COVID-19 mAb treatment, the combination of casirivimab and imdevimab, which mediates infection of Raji and THP-1 cells by pseudoparticles composed of SARS-CoV-2 spike protein [46].

In vivo assessment of ADE is highly dependent upon the availability of a suitable animal model that is permissive of viral replication to levels similar to those observed in humans, as well as displaying clinical and histopathological correlates of disease. Additionally, animal model suitability must also be related to its ability to recapitulate human Fc receptor biology. Rodent and ferret models have been useful for elucidating mechanisms of SARS-CoV-2 pathogenesis and quickly screening countermeasures but have limited value in terms of modeling host responses specific to humans. We therefore used AGMs in our in vivo studies, as this model was previously described to mirror the main features of SARS-CoV-2 infection in humans and also possesses common Fc receptor activity [31,32,34].

The pathology findings in all treatment groups were consistent with previous publications of experimental SARS-CoV-2 infection in AGMs, including varying severities of broncho-interstitial pneumonia induced by SARS-CoV-2 [31,32,34]. Minimal-to-mild attenuation in the severity of infection was observed in AGMs treated with 0.05 mg/kg (clinical pathology only) and 20 mg/kg (clinical and anatomic pathology) of bamlanivimab when compared with the isotype control groups. While biologic variation could not be definitively excluded as a contributor to these observed differences, our findings are compatible with a test article-related effect. Bamlanivimab treatment at a sub-saturating or saturating dose prior to inoculation with SARS-CoV-2 did not promote ADE of SARS-CoV-2 infection as measured by viral load in nasal passages, bronchoalveolar lavage, and lung tissue. No evidence of exaggerated inflammation or exaggerated SARS-CoV-2 antigen consistent with histologic evidence of ADE was noted in pulmonary tissues. Immunohistochemical labeling of lung tissue for viral antigen for SARS-CoV-2 was absent in the highest dose of bamlanivimab with no appreciable difference in labeling in sub-saturating dose and isotype control monkeys. Additionally, lesion severity was similar across treatment and control groups.

One AGM in the sub-saturating bamlanivimab group was humanely euthanized one day prior to scheduled sacrifice due to clinical signs. Notably, this AGM had elevated IL-6 levels, which were comparable to fatal levels in AGMs observed in a recent study [32]. Although this single AGM exhibited severe symptoms, no other AGMs that received the same sub-saturating concentration of the antibody exhibited any disease enhancement. Additionally, the viral loads measured in this single AGM were no greater than in the control animals, which therefore suggested that the underlying cause of pathophysiology was host-dependent rather than due to bamlanivimab treatment. This conclusion is supported by recent non-peer-reviewed data indicating that host specific differences may influence disease outcomes [42]. Moreover, while disease presentation of SARS-CoV-2 infection in monkeys is generally mild, severe disease leading to morbidity and death has been previously observed in AGMs [32].

Overall, bamlanivimab was well tolerated regardless of dose. The AGM model of SARS-CoV-2 infection demonstrated anticipated clinical signs consistent with the viral infection. Virology determinations confirmed infections in all animals and demonstrated significant neutralization at the 20 mg/kg IV dose of bamlanivimab as compared to controls. It is also noteworthy that while the lower concentration of bamlanivimab was sub-saturating and led to sub-optimal neutralization, there was a significant decrease in viral loads in nasal swabs and some lung tissues, confirming the potency of bamlanivimab. This is the report of sub-saturating doses of bamlanivimab exhibiting neutralizing activity. In summary, bamlanivimab potently inhibited viral replication in the nasal passages, pharynx, and lungs of AGMs at sub-saturating and saturating concentrations. It does not lead to enhanced viral loads, enhancement of disease nor dissemination outside of the upper and lower respiratory tract.

As with all preclinical studies, there are limitations in translating this work into the clinical setting, as it is well established that in vitro and in vivo models of ADE may not fully translate to the clinic. As the advanced pathology of SARS-CoV-2 is similar to that observed in ADE, more data are required to distinguish the causative factor. However, there is currently no clear clinical evidence that anti-SARS-CoV-2 therapies induce ADE. Recent non-peer-reviewed data from 13,127 patients in the RECOVERY trial showed that there was no significant difference in 28-day mortality between patients receiving convalescent plasma versus allocated usual care [47]. In addition, clinical trials of multiple vaccines have not reported vaccine-associated enhanced respiratory disease [48,49,50]. This study assessed a single mAb. However, the clinical safety profile of bamlanivimab is similar to other mAbs, with and without Fc modifications that nullify effector function, thereby indicating that ADE is not occurring [2,51].

## 5. Conclusions

This study contributes to the current understanding of whether COVID-19 mAbs may exacerbate SARS-CoV-2 infection through ADE both in vitro and in vivo. To summarize, our in vitro data demonstrated that bamlanivimab, which possesses expected effector function, does not enhance SARS-CoV-2 infection of Fc receptor-expressing cell lines or primary human macrophages. Similarly, our in vivo data using an NHP model also demonstrated that enhanced SARS-CoV-2 infection was not observed in the presence of bamlanivimab. Altogether, our findings suggest that bamlanivimab, even at low sub-neutralizing concentrations, poses a low risk of ADE in patients and further supports the safety profile of monoclonal antibodies as a treatment of COVID-19.

## Figures and Tables

**Table 1:** Summary of select lung microscopic findings at terminal euthanasia on day 5.

	Incidence (Total Lobes Affected)							
	Male				Female			
	Control		Bamlanivimab		Control		Bamlanivimab	
Dose (mg/kg)	0.05	20	0.05	20	0.05	20	0.05	20
**Inflammation, Mixed Cell, Bronchi/Bronchioles**								
Minimal	3	3	8	2	7	2	3	0
Mild	11	10	2	10	2	6	10	7
Moderate	3	5	4	1	1	1	2	0
Marked	1	0	0	0	0	0	0	0
**Increased Cells, Alveolar/Septal**								
Minimal	2	2	1	3	4	1	0	4
Mild	12	13	10	15	12	10	17	14
Moderate	4	1	5	0	0	1	0	0
Marked	0	0	0	0	1	0	0	0
**Congestion/Hemorrhage, Alveolar**								
Minimal	8	7	3	8	0	3	3	4
Mild	2	7	8	2	6	2	12	8
Moderate	0	3	5	0	4	0	0	0
Marked	0	0	0	0	2	1	0	0

*N* = 3 for all groups except control (20 mg/kg) where *N* = 2.

## Data Availability

Additional details relevant to materials, methods, and datasets used in this manuscript are available from the corresponding author upon reasonable request.

## Supplementary Materials

The following supporting information can be downloaded at: https://www.mdpi.com/article/10.3390/pathogens12121408/s1, Table S1: In vitro binding parameters of bamlanivimab to human Fcγ receptor ECDs measured using surface plasmon resonance at 25 °C; Figure S1: Binding affinities to Fc gamma receptors; Figure. S2: Quantification of viral RNA in Vero-E6 and ST486 cells at 6- and 48-h postinfection (hpi); Figure S3: Quantification of viral RNA in Raji, THP1 and primary human macrophage at 6-h post-infection (hpi); Figure S4: Quantification of proinflammatory cytokine markers from AGMS treated with bamlanivimab (varying doses), challenged with SARS-CoV-2.

## Abbreviations

ACE2: angiotensin-converting enzyme 2; ADCC: antibody-dependent cellular cytotoxicity; ADCP: antibody-dependent cellular phagocytosis; ADE: antibody-dependent enhancement; AGM: African green monkey; ALP: alkaline phosphatase; ALT: alanine aminotransferase; AST: aspartate aminotransferase; BAL: bronchoalveolar lavage; CDC: complement-dependent cytotoxicity; CHO: Chinese hamster ovary; *C_T_*: threshold cycle; DENV: dengue virus; DPBS: Dulbecco’s HyClone; DPI: days post infection; ECD: extracellular domain; EUA: Emergency Use Authorization; FcγR: Fc gamma receptors; FDA: Food and Drug Administration; GEq: genome equivalents; GGT: gamma-glutamyl transferase; hpi: hours post infection; IHC: immunohistochemical; i.n.: intranasal; i.t.: intratracheal; i.v.: intravenously; KD: affinity; LLOQ: lower limit of quantification; mAbs: monoclonal antibodies; MEM: minimum Essential Medium Eagle; MERS: Middle East respiratory syndrome; MMRM: mixed model repeated measures model; N: nucleoprotein; NEAA: non-essential amino acids; NHP: non-human primates; NTD: N-terminal domain; PBS: phosphate-buffered saline; PFU: plaque-forming units; PRNT_50_: 50% of viral plaques; RT-qPCR: reverse transcriptase-polymerase chain reaction; RBD: receptor binding domain; RMSE: residual error term; RT: room temperature; UTMB: University of Texas Medical Branch; SARS-CoV-2: severe acute respiratory syndrome coronavirus-2.
