# Supplementary material for: The Therapeutic Monoclonal Antibody Bamlanivimab Does Not Enhance SARS-CoV-2 Infection by FcR-Mediated Mechanisms
Source: Pathogens. 2023 Nov 30;12(12):1408. doi: 10.3390/pathogens12121408 (PMC10746090; doi:10.3390/pathogens12121408)

Supplementary Material

Supplementary Tables and Figures

Tables

Table S1: *In vitro* binding parameters of bamlanivimab to human Fcγ receptor ECDs measured using surface plasmon resonance at 25˚C

Figures:

Figure S1: Binding affinities to Fc gamma receptors

Figure S2: Quantification of viral RNA in Vero-E6 and ST486 cells at 6- and 48-hours post-infection (hpi)

Figure S3: Quantification of viral RNA in Raji, THP1 and primary human macrophage at 6-hours post-infection (hpi)

Figure S4: Quantification of proinflammatory cytokine markers from AGMS treated with bamlanivimab (varying doses), challenged with SARS-CoV-2

Table S1: In vitro binding parameters of bamlanivimab to human Fcγ receptor ECDs measured using surface plasmon resonance at 25˚C

Sample	Human Ligand	Average KD	Std Dev
**LSN2436595**	Fcγ R1	58.9 pM	5.4
**LSN2835015**	Fcγ R1	189.8 nM	19.2
**Bamlanivimab**	Fcγ R1	68.0 pM	6.3
**LSN2436595**	Fcγ RIIA_131H	0.67 µM	0.06
**LSN2835015**	Fcγ RIIA_131H	>10 µM	
**Bamlanivimab**	Fcγ RIIA_131H	0.70 µM	0.06
**LSN2436595**	Fcγ RIIA_131R	0.64 µM	0.02
**LSN2835015**	Fcγ RIIA_131R	>10 µM	
**Bamlanivimab**	Fcγ RIIA_131R	0.68 µM	0.02
**LSN2436595**	Fcγ RIIb	3.24 µM	0.03
**LSN2835015**	Fcγ RIIb	>10 µM	
**Bamlanivimab**	Fcγ RIIb	3.30 µM	0.05
**LSN2436595**	Fcγ RIIIA_158V	0.14 µM	0.01
**LSN2835015**	Fcγ RIIIA_158V	7.13 µM	0.16
**Bamlanivimab**	Fcγ RIIIA_158V	0.15 µM	0.01
**LSN2436595**	Fcγ RIIIA_158F	0.93 µM	0.02
**LSN2835015**	Fcγ RIIIA_158F	>10 µM	
**Bamlanivimab**	Fcγ RIIIA_158F	0.87 µM	0.04

Note: LSN2436595, IgG1 positive control antibody; LSN2835015, human IgG4 PAA negative control antibody n ≥ 3x assayed. pM, pico molar; Std Dev, standard deviation; µM, micro molar

Figure S1: Binding affinities to Fc gamma receptors

(A) Expression of Fc gamma receptors on the ST486, Raji (B-lymphocytic), THP-1 (monocytic) human cell lines and (B) expression of FcγRs on human primary macrophages from three representative donors following 24 hours plating. Cells were detached with Accutase and 30,000 cells were then left unstained, stained for FcγR or for corresponding isotype controls and read on the BD LSR Fortessa II. Dead cells, debris, and doublets were excluded from flow analysis. Data for THP1 and Raji cell lines (from one experiment, out of two experiments) and ST486 (tested once) plotted as histograms.

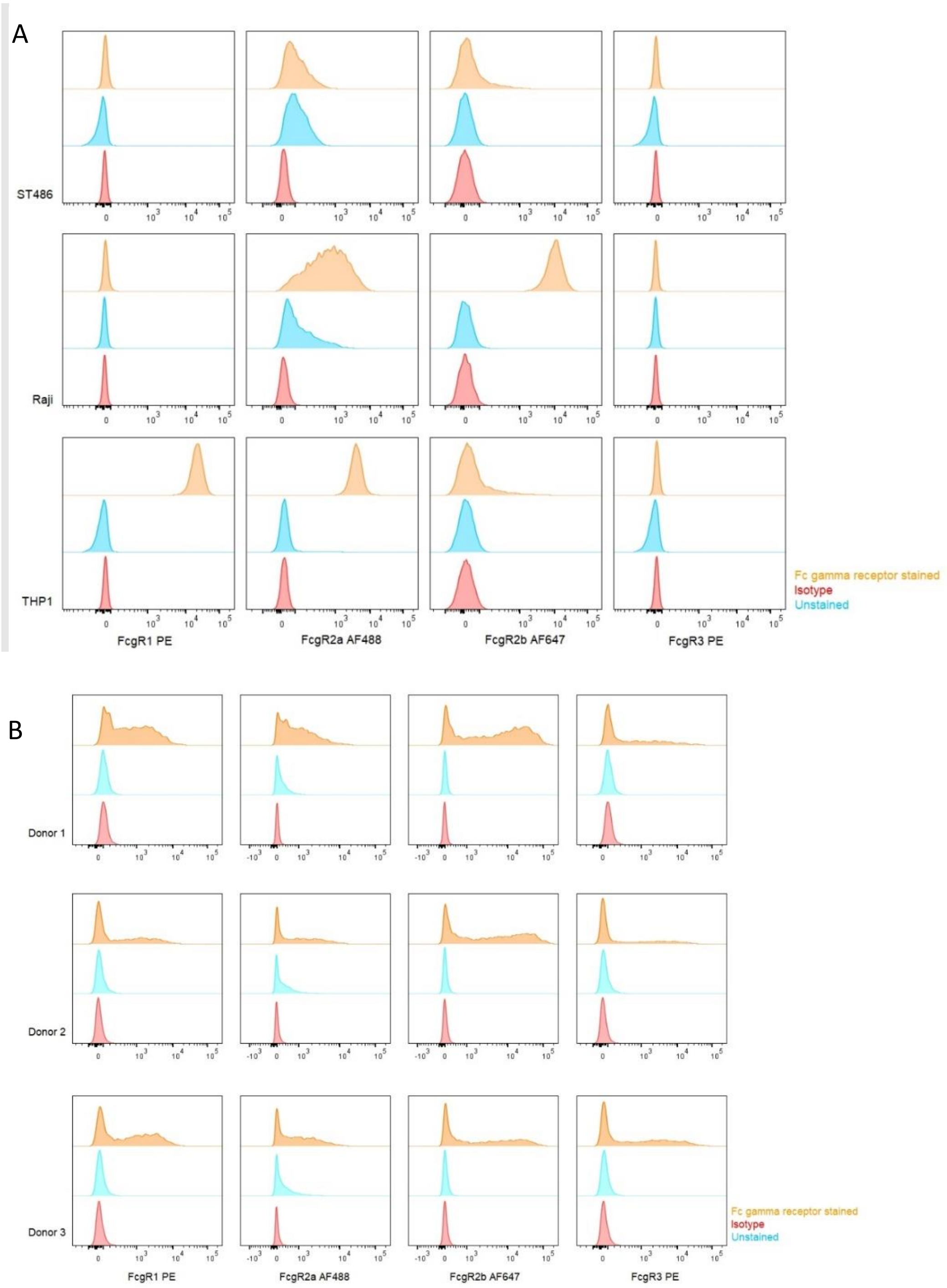

Figure S2: Quantification of viral RNA in Vero-E6 and ST486 cells at 6- and 48-hours post-infection (hpi)

Intracellular positive-strand RNA, intracellular negative-strand RNA and extracellular viral RNA in Vero-E6 at 6 hpi (a-c) and 48 hpi (d-f) and ST486 cells at 6 hpi (g-i) and 48 hpi (j-l). Cells were incubated with SARS-CoV-2 at 0.1 MOI in the presence or absence of bamlanivimab (0.3 – 3000 ng/mL) or IgG1 isotype control (0.3 – 3000 ng / mL). Bars denote mean +/- SD (N=3 per sample). GE, genome equivalents (LOG10) of SARS-CoV-2 in cells at 6 hpi with the virus at an MOI of 0.1.

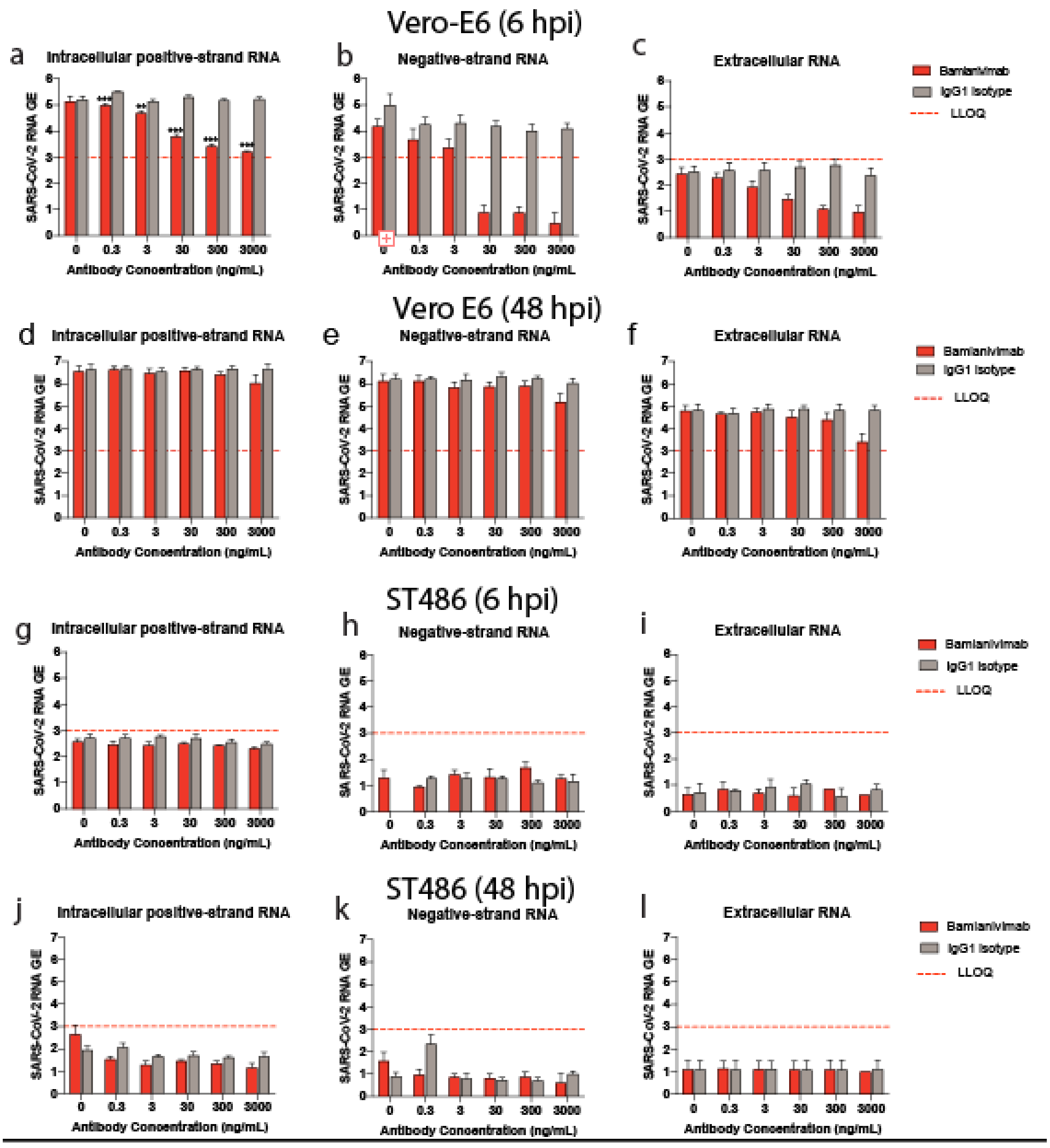

Figure S3: Quantification of viral RNA in Raji, THP1 and primary human macrophage at 6-hours post-infection (hpi)

Intracellular positive-strand RNA, intracellular negative-strand RNA and extracellular viral RNA in Raji cells (a-c), THP1 cells (d-f) and representative primary human macrophages (g-i) at 6 hpi. Cells were incubated with SARS-CoV-2 at 0.1 MOI in the presence or absence of bamlanivimab (0.3 – 3000 ng/mL) or IgG1 isotype control (0.3 – 3000 ng / mL). Bars denote mean +/- SD (N=3 per sample). GE, genome equivalents (LOG10) of SARS-CoV-2 in cells at 6 hpi with the virus at an MOI of 0.1.

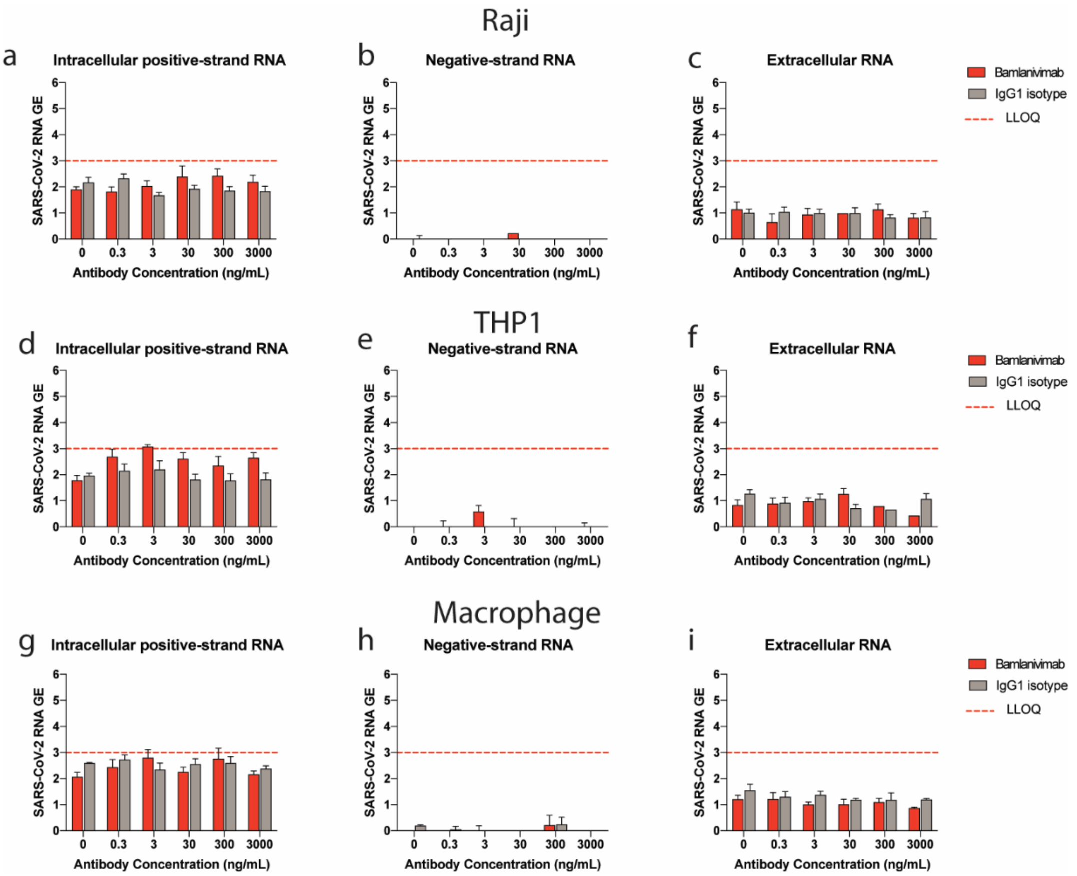

Figure S4: Quantification of proinflammatory cytokine markers from AGMS treated with bamlanivimab (varying doses), challenged with SARS-CoV-2

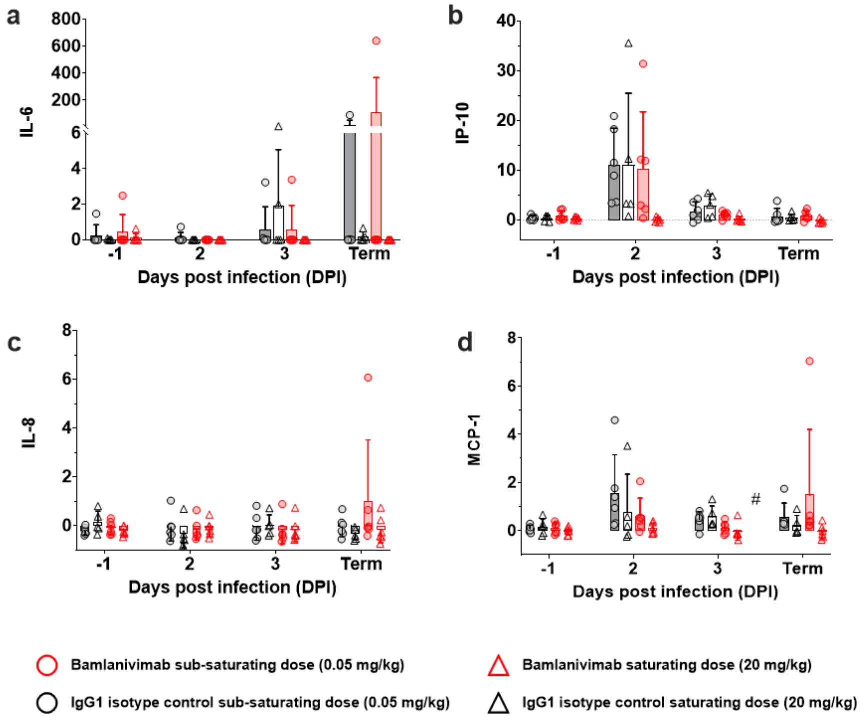

Supplement: Supplementary file 1 [file pathogens-12-01408-s001.zip › pathogens-2656436-supplementary.pdf]
